# Serine Utilization by *Campylobacter Jejuni* and *Campylobacter Coli*

**DOI:** 10.14252/foodsafetyfscj.D-25-00004

**Published:** 2025-09-12

**Authors:** Ayako Watanabe-Yanai, Taketoshi Iwata, Yukino Tamamura-Andoh, Nobuo Arai, Anna Momoki, Masahiro Kusumoto

**Affiliations:** 1 Division of Zoonosis Research, National Institute of Animal Health, National Agriculture and Food Research Organization, 3-1-5 Kannondai, Tsukuba, Ibaraki 305-0856, Japan; 2 Graduate School of Veterinary Science, Osaka Metropolitan University, 1-58 Rinku-oraikita, Izumisano, Osaka 598-8531, Japan

**Keywords:** amino acids, *Campylobacter jejuni*, *Campylobacter coli*, serine, proliferative ability

## Abstract

The intestinal pathogens *Campylobacter jejuni* and *Campylobacter coli* are the most common causes of foodborne bacterial gastroenteritis in humans. In Japan and globally, more than 90% of *Campylobacter* species isolated from patients with *Campylobacter* food poisoning are *C. jejuni*, whereas *C. coli* accounts for only a small percentage. This difference in isolation rates is considered to be due to differences in the ability of *C. jejuni* and *C. coli* to proliferate within the host. However, only a few studies have compared the growth of these two pathogens. To investigate the effect of *C. jejuni* on the proliferative ability of *C. coli* and vice versa, co-culture experiments were conducted. Similar strains were selected on the basis of their amino acid requirements for comparative purposes. *C. jejuni* grew on amino acid-rich media, indicating that its growth was not affected by the presence of *C. coli*. By contrast, the growth of *C. coli* was inhibited by *C. jejuni*. This suggests that the higher detection rate of *C. jejuni* may be due to its superior growth capacity rather than its initial abundance. Further research on *C. coli* is required to better understand its role and behavior in the host and in different environments.

## 1. Introduction

**Fig. 1.**
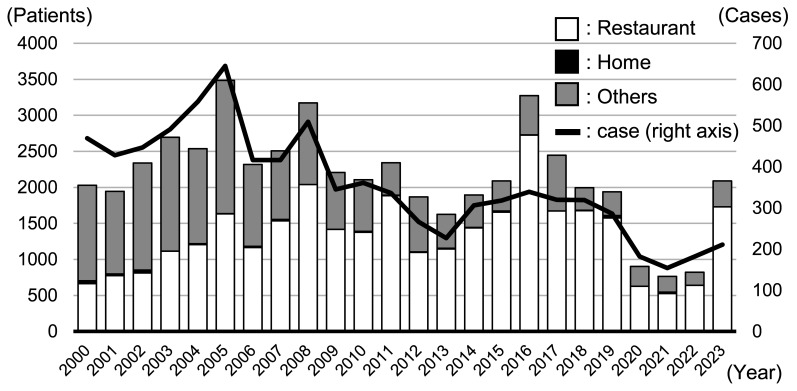
Number of *Campylobacter* foodborne disease cases and patients in Japan from 2000 to 2023 The number of cases of *Campylobacter* food poisoning is represented by the line graph, and the number of patients is represented by the bar graph. Adopted from statistical data by the Ministry of Health, Labour and Welfare of Japan^1^^)^.

Campylobacteriosis, a foodborne disease attributed to *Campylobacter* spp., is a leading cause of bacterial gastroenteritis worldwide. According to data compiled by the Ministry of Health, Labour and Welfare on incidences of foodborne diseases in Japan, 200–300 cases of campylobacteriosis occur annually, affecting approximately 2,000 patients^1^^)^ (**Fig. 1**). Epidemiological studies on campylobacteriosis in Japan have shown that more than 80% of cases occurred in restaurants, with only a few occurring in homes, and raw and undercooked poultry and meat products were the main causes^1^^)^. Additionally, the decrease in campylobacteriosis cases between 2020 and 2022 is possibly due to the stagnation in economic activity resulting from the COVID-19 pandemic. The number of patients afflicted by campylobacteriosis in restaurants decreased between 2020 and 2022 (**Fig. 1**) coinciding with a reduction in dining-out opportunities due to voluntary restrictions on going out. However, after the stay-at-home orders had been lifted, the number of patients who acquired the disease in restaurants in 2023 reached levels similar to those before the 2019 restrictions (**Fig. 1**). By contrast, the number of patients affected in the home environment did not change after the COVID-19 pandemic (**Fig. 1**). Thus, the highly fluctuating number of patients with campylobacteriosis in Japan was largely due to patrons who acquired the disease in restaurants^1^^)^.

Campylobacteriosis is the principal cause of bacterial diarrhea in humans. *C. jejuni* and *C. coli* are the two most common etiological agents of this disease, with 75% of cases caused by the former, approximately 10% by the latter, and approximately 14% caused by either one of them but unable to be distinguished and 1% caused by other *Campylobacter* spp.^2^^,^^3^^)^. This ratio is a result of the culture method and thus depends on the number of viable bacteria isolated from infected patients. That is, this ratio could reflect differences in the abilities of *C. jejuni* and *C. coli* to grow in the human gastrointestinal tract rather than initial abundance in this environment.

In general, the ability of *C. jejuni* to survive and thrive in a wide range of environmental niches is determined by its ability to utilize different metabolites available in different hosts and environments. In contrast to the many studies on *C. jejuni*, only a few reports on the metabolism of *C. coli* exist in the literature. It is well recognized that amino acids are important carbon and energy sources for *C. jejuni*, both in vitro and in vivo. The primary nutrient sources for *C. jejuni* are serine, aspartate, asparagine, and glutamate^4^^)^. *C. jejuni* strain NCTC 11168 grows well in nutrient-restricted media, with aspartate, glutamate, proline, or serine as the main energy sources^5^^)^. In particular, serine is an important growth nutrient for *C. jejuni* NCTC 11168^6^^)^. Additionally, a novel L-fucose pathway involving L-fucose permease has been shown to exist within the genomic island of certain *C. jejuni* strains, indicating that some strains of this species can metabolize L-fucose^7^^)^. Likewise, some strains of *C. coli* are able to metabolize L-fucose^7^^)^. Additionally, *C. coli* can use propionate as a carbon source owing to its possession of the propionate-CoA ligase and 2-methyl-synthase genes, which are absent in *C. jejuni*^8^^)^. Propionic acid is found at high concentrations in the gastrointestinal tract of pigs^9^^)^, which are among the natural hosts of *C. coli*; this may offer *C. coli* a selective benefit in colonizing the gastrointestinal tract of pigs. However, reports on amino acid metabolism in *C. coli* are lacking.

Therefore, the objective of this study was to examine the amino acid requirements of *C. coli*. Serine was selected as an important factor to explain the difference in growth characteristics between *C. jejuni* and *C. coli*. We found that several *C. coli* strains isolated from healthy broiler chickens had high serine requirements. Furthermore, in a co-culture of these two species in serine-containing medium, *C. coli* did not affect the growth of *C. jejuni*, whereas *C. jejuni* did affect the growth of *C. coli*. The serine requirement of *C. coli* may cause the bacterium to grow less well than *C. jejuni* under coexisting conditions. Further research is required to establish whether this introduces bias into investigations of the causes of campylobacteriosis.

## 2. Materials and Methods

### 2.1. Growth Assay

The strains used in this study are listed in **Table 1**. For *C. jejuni*, we used NCTC 11168 as the reference strain and six other strains isolated from broilers in Japan. For *C. coli*, we used BAA-1061 as the reference strain and six other strains isolated from broilers in Japan. The strains were selected from the most prevalent sequence types in the PubMLST database: ST-21 Clonal Complex (22.2%) for *C. jejuni* and ST-828 Clonal Complex (82.8%) for *C. coli*^10^^)^. Minimum Essential Medium (MEM) M0275 (Sigma‒Aldrich, USA) supplemented with Fe^2+^ in the form of iron(II)-ascorbate (FUJIFILM Wako Pure Chemical Co., Ltd., Japan) was used for the growth assays. This medium did not contain serine, aspartate, glutamate, or proline, which are important nutrient sources for *C. jejuni* and *C. coli*. To assess the importance of these four amino acids for *C. jejuni* growth, two different media were prepared: a basal medium (MEM containing 10 mM each of serine, aspartate, glutamate, and proline) and the basal medium without serine. *C. jejuni* and *C. coli* were grown separately to the log phase in Mueller–Hinton (MH) broth, after which the cells were pelleted, washed once, resuspended in the abovementioned media at an OD_600_ of 0.0005 (around 10^5^ CFU/mL), and incubated at 42 °C for 20 h under microaerophilic conditions with orbital shaking (130 rpm). All assays were performed in triplicates in three independent experiments.

**Table 1.** Bacterial strains in this study

Bacterial species	Strain	Source	Sequence type (Clonal complex)
*Campylobacter jejuni*	NCTC 11168	Human	43 (21)
	CJ001	Chicken	21 (21)
	CJ002	Chicken	2789 (21)
	CJ003	Chicken	4253 (21)
	CJ004	Chicken	4526 (21)
	CJ005	Chicken	4526 (21)
	CJ006	Chicken	4526 (21)
*Campylobacter coli*	ATCC BAA-1061	Chicken	1063 (28)
	CC001	Chicken	854(828)
	CC002	Chicken	854 (828)
	CC003	Chicken	854 (828)
	CC004	Chicken	1761 (828)
	CC005	Chicken	1767 (828)
	CC006	Chicken	4172 (828)

### 2.2 Co-culture Growth Assay

Co-cultivation studies were performed with *C. jejuni* NCTC 11168 and *C. coli* CC005, which have similar growth and serine requirements. MEM containing 10 mM each of serine, aspartate, glutamate, and proline was used. The two strains were grown separately to the log phase in MH broth, after which the cells were pelleted, washed once, resuspended in the abovementioned media at an OD_600_ of 0.00005 (around 10^4^ CFU/mL), and incubated at 42 °C under microaerophilic conditions in static culture. Then, the two strains were mixed at a ratio of 1:1, and colonies were measured after 0, 6, 24, and 48 h of incubation at 42 °C under microaerophilic conditions in static culture. The bacterial growth was monitored by counting the number of colonies grown on MH agar plates under microaerophilic conditions at 42 °C for 48 h. Because *C. coli* CC005 is tetracycline resistant, a medium supplemented with tetracycline (FUJIFILM Wako Pure Chemical Co., Ltd.) was used for colony counting of the strain. We determined the tetracycline concentration used in this test after confirming that it did not affect the growth of *C. coli* CC005. To estimate the number of colony-forming units (CFU) of *C. jejuni*, the number of *C. coli* CC005 colonies was subtracted from the total number of colonies on MH agar. All assays were performed in triplicates in three independent experiments.

### 2.3 Statistical Analysis

Statistical analyses were performed using EZR version 1.61 (November 11, 2022)^11^^)^. Comparisons between independent groups were performed using Student’s *t*-test, whereas multiple group comparisons were performed using one-way analysis of variance.

## 3. Results

### 3.1 Serine Dependence of *C. jejuni*

**Fig. 2.**
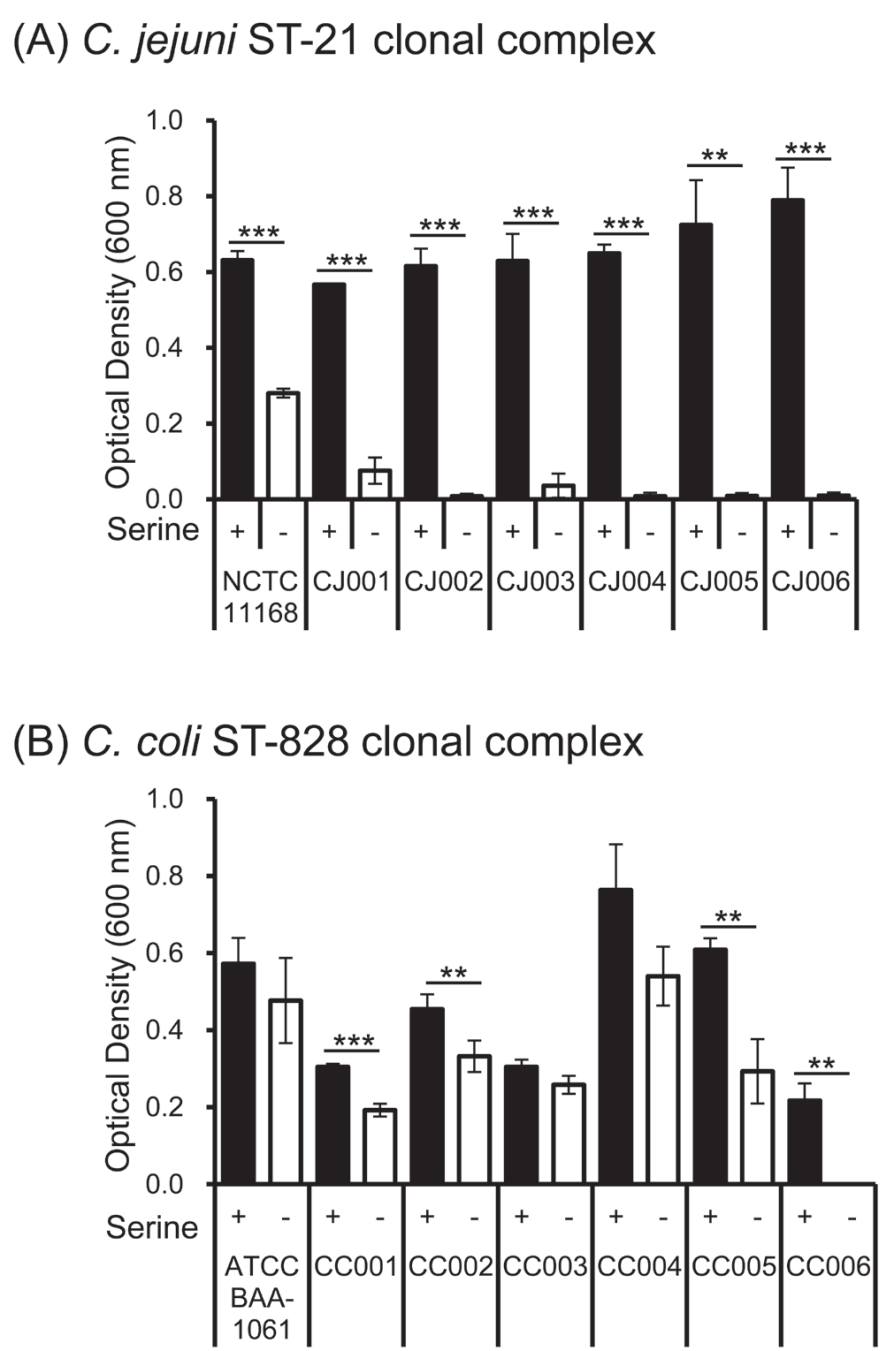
Serine utilization by *C. jejuni* and *C. coli* strains Growth of *C. jejuni* (A) and *C. coli* (B) in different combinations of amino acids. “+” and “-” under the x-axis indicate the culture medium with and without serine amendment, respectively. After 24 h, growth was scored by measuring the turbidity at OD_600 nm_. Values are the mean and standard error from three independent experiments. **p < 0.01, ***p < 0.001.

The reference strain *C. jejuni* NCTC 11168 and all six *C. jejuni* strains isolated from broiler chickens showed significantly decreased proliferation in the serine-deficient medium compared with that in the serine-supplemented basal medium (**Fig. 2A**).

### 3.2 Serine Dependence of *C. coli*

The reference strain *C. coli* BAA-1061 and two of the strains isolated from broiler chickens (CC003 and CC004) showed no significant differences in proliferation in the presence or absence of serine (**Fig. 2B**). By contrast, the other four strains (CC001, CC002, CC005, and CC006) showed significantly inhibited proliferation in serine-deficient medium (**Fig. 2B**).

### 3.3 Effect of *C. jejuni* on the Proliferation of *C. coli*

**Fig. 3.**
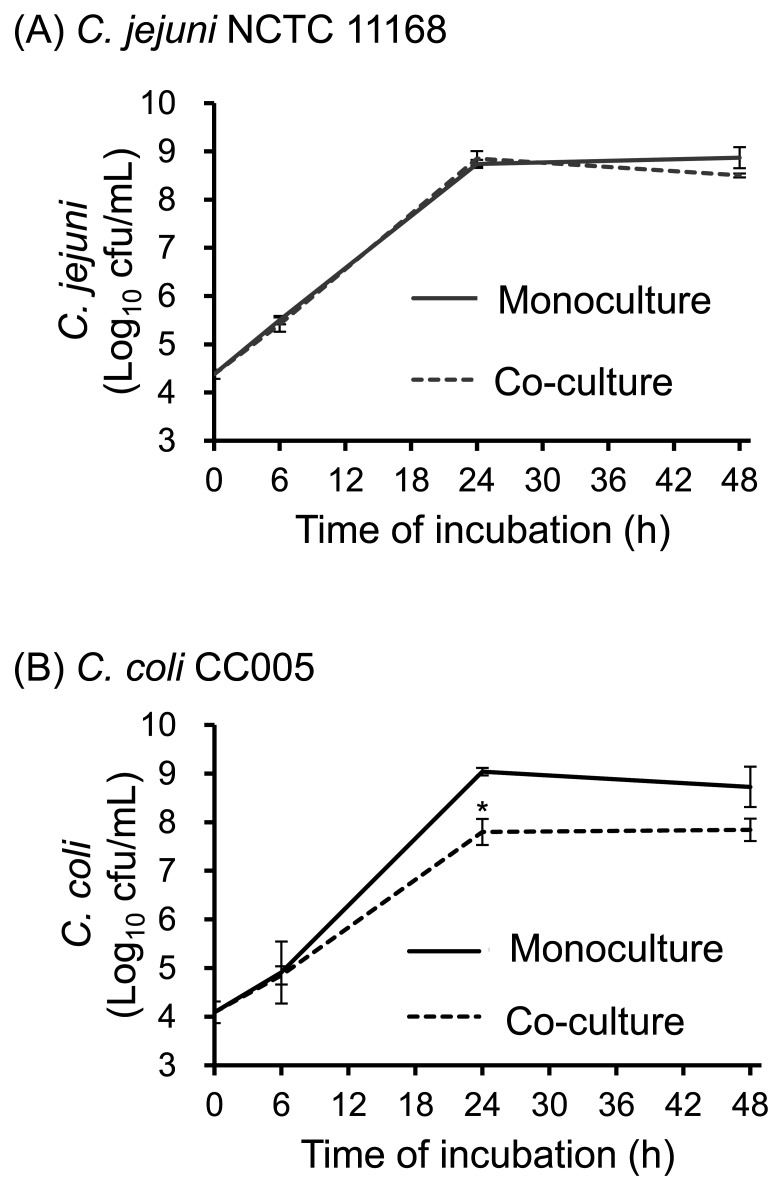
Comparison of *C. jejuni* NCTC 11168 and *C. coli* CC005 bacterial counts over time in monoculture and co-culture Growth curves of *C. jejuni* NCTC 11168 (A) and *C. coli* CC005 (B). *C. jejuni* NCTC 11168 and *C. coli* CC005 were each cultured separately (solid line), and a co-culture experiment was performed by mixing both strains at a 1:1 ratio (dashed line). The medium in this experiment was supplemented with four amino acids, and colonies were measured after 0, 6, 24, and 48 h of incubation. Values are the mean and standard error from three independent experiments. *p < 0.05.

*C. jejuni* NCTC 11168 proliferation in monoculture (solid line) did not differ significantly from that in co-culture (dashed line) (**Fig. 3A**). By contrast, the bacterial count of *C. coli* CC005 after 24 h in co-culture with *C. jejuni* (dashed line) was significantly lower than that in the monoculture (solid line) (**Fig. 3B**).

## 4. Discussion

*C. jejuni* is often isolated from chickens, which are rich in amino acids (including serine)^12^^,^^13^^)^, whereas *C. coli* is commonly isolated from pigs, which are rich in propionic acid^9^^)^. There is no doubt that amino acids are important for the growth and metabolism of *C. jejuni*, with serine in particular playing an important role (**Fig. 2A**). At the same time, this study revealed that some strains of *C. coli* also require amino acids for proliferation (**Fig. 2B**), a novel finding that has not been clarified in previous studies^7^^,^^8^^)^. These results indicate a high diversity of nutrient requirements among *C. coli* strains. However, the interpretation of these findings requires further validation.

The significantly inhibited growth of *C. coli* strains with serine requirements is possibly due to nutrient competition with *C. jejuni* (Fig. 3B). This suggests that when identifying the pathogens in samples from campylobacteriosis patients, the involvement of *C. coli* may be underestimated. In Japan, both *C. jejuni* and *C. coli* have been reported as causative agents of campylobacteriosis, with *C. jejuni* estimated to account for more than 70% of cases^1^^)^. Therefore, *C. coli* is generally considered less likely than *C. jejuni* to cause this disease. However, this study suggests that the density ratios of *C. jejuni* and *C. coli* isolated from patients may not be indicative of their actual ratio in the host. That is, this ratio may simply reflect differences in the abilities of *C. jejuni* and *C. coli* to proliferate in a gastric environment with high amino acid concentrations rather than the number of viable bacteria in the human gastrointestinal tract. Factors that determine the proliferative ability of bacteria include genetic, nutritional, and environmental aspects. However, because research on *C. coli* has lagged behind that on *C. jejuni*, the factors that most affect *C. coli* growth remain unknown.

In conclusion, we have shown the existence of *C. coli* strains that, like *C. jejuni*, utilize serine as an energy source. Also, *C. coli* had previously been regarded to use a different energy source to that used by *C. jejuni*. Furthermore, *C. coli* growth was inhibited by *C. jejuni* when both species were co-cultured under serine-sufficient conditions. Therefore, because the growth of *C. coli* is affected by *C. jejuni*, the existing culture methods may not provide an accurate estimate of the exact initial abundance of *C. coli* in mixed samples of the two species. Further analysis of the mechanisms underlying the amino acid requirements of *C. coli* is expected to elucidate its ability to proliferate in an amino acid-rich environment such as the human gastrointestinal tract, leading to an accurate assessment of its contribution to campylobacteriosis.
